# Comparing the Effect of Foot Massage with Grape Seed Oil and Sweet Almond Oil on Physiological Leg Edema in Primigravidae: A Randomized Clinical Trial

**DOI:** 10.1155/2020/6835814

**Published:** 2020-01-13

**Authors:** Maryam Navaee, Marzieh Rakhshkhorshid

**Affiliations:** Midwifery Department, Pregnancy Health Research Centre, Zahedan University of Medical Sciences, Zahedan, Iran

## Abstract

**Background:**

Leg edema is a prevalent problem in pregnancy causing activity restrictions for pregnant women. This study was performed to compare the effect of foot massage using grape seed oil and sweet almond oil on physiological leg edema.

**Methods:**

A randomized clinical trial was conducted on 90 primigravidae referred to public health centres of Zahedan, Iran. The participants' gestational age was 30–40 weeks. The study was conducted from August 2016 to November 2017. The participants were randomly assigned to three groups (massage with grape seed oil, massage with sweet almond oil, and without intervention). After determining the extent of leg edema, foot massages were done for 20 minutes within 5 days in the two intervention groups. Then, foot circumferences were measured on day 5 after the intervention. Foot circumferences for the control group were measured on days 1 and 5. A nonelastic tape measure was used to measure the circumferences. To analyse the data, SPSS 21 software and statistical tests including one-way ANOVA, Tukey's test, and paired *t*-test were used.

**Results:**

The results from this study showed a significant difference in the mean score change of foot circumferences between groups (*P*=0.001). According to the results of Tukey's test, mean score changes of foot circumferences of both intervention groups were significantly different those of the control group. However, this difference was not significant between the two intervention groups (*P*=0.865).

**Conclusion:**

The findings of this study confirmed the effectiveness of foot massage using grape seed and sweet almond oils to reduce pregnancy physiological edema. Therefore, foot massage with appropriate oils can be used as a useful technique by trained midwives in prenatal care centres or at pregnant women houses. This trial is registered with IRCT2015072723370N1.

## 1. Introduction

Anatomic and physiologic changes caused by pregnancy result in an array of symptoms affecting the lower extremity [1, 2]. The increasing weight of the growing uterus puts pressure on pelvic veins and on the inferior vena. This increases blood pressure in leg veins leading to venous insufficiency and leg edema. Leg edema can affect up to 80% of pregnant women and should not be considered a sign of pregnancy-induced hypertension or preeclampsia [3]. The most common symptom of edema is the experience of substantial pain, as well as night cramps, numbness, and tingling. In addition, legs may feel heavy and achy and possibly be unsightly [4]. Symptoms tend to worsen after long periods of standing and with each successive pregnancy [3]. Since treatments to relieve common discomforts of pregnancy such as edema should not threaten the mother and fetus [5–7], many midwives and pregnant women seek complementary therapies such as massage therapy [3, 8]. A nonpharmacological intervention which is popular among healthcare providers is foot massage [9]. The technique in leg and foot edema works by moving extravascular fluid without disturbing intravascular fluid [10], increasing peripheral blood flow, boosting oxygen, and therefore reducing edema [11].

Using oil during a massage helps the hands of a masseur move easily on the skin and causes the skin to minimize dryness [12]. Grape seed oil and sweet almond oil are used in therapeutic massage [13]. These oils are good sources of essential fatty acids and vitamin E, polyphenols, and flavonoids [14] and cause some type of improvement in varicose veins, hemorrhoids, and limb edema by improving blood circulation. In addition to their anti-inflammatory properties, they can be effective in relieving edema caused by problems such as rheumatoid arthritis [15]. Sweet almond oil contains vitamin E and riboflavonoids that, of course, have little antioxidant effect [16]. However, its advantage compared to grape seed oil is that it is cheaper and more accessible [17, 18].

In general, studies on the effects of massage therapy on a variety of healthcare settings have shown that this therapy could be used as an effective treatment in a series of discomforts while causing no damage [19]. Çoban and Şirin in their study on the effect of foot massage with grape seed oil on edema during pregnancy found significant results in reducing edema and that the reduction was greater in the right leg [20]. In a study conducted by Rahimikian et al. on the effect of foot massage on physiological edema with baby oil, significant results were seen in reducing edema [21].

Since, to our knowledge, few studies have examined the effects of foot massage on edema resulting from pregnancy and no studies have compared the results of using different oils to select the best oils in massage, this study was performed with the aim to “compare the effect of foot massage with grape seed oil and sweet almond oil on physiological leg edema during pregnancy.”

## 2. Materials and Methods

This randomized clinical trial study was conducted on a number of primigravidae referred to health centres in Zahedan, Iran, from August 2016 to November 2017. The sample size was calculated using the following formula (equation (1)) based on a previous study [22]:

(1) $$\begin{array}{c} \text{sample size }=\;\frac{{\left(z_{1}-\left(\alpha /2\right)+z_{1}-\beta \right)}^{2}\left(s_{1}^{2}+s_{2}^{2}\right)}{{\left({\bar{x}}_{1}-{\bar{x}}_{2}\right)}^{2}}. \end{array}$$

Using a power of 90% and taking into account the 95% confidence interval, the required sample size was calculated to be 24 for each group (${\bar{x}}_{1}$ = 4/7, ${\bar{x}}_{2}$ = 3/3, *s*_1_ = 1/7, and *s*_2_ = 1/2). Assuming 10% for attrition, 30 primigravidae were needed for each group.

In this study, a multistage sampling technique was used. First, the city was divided into five geographical regions including the north, south, east, west, and central. Five health centres were chosen randomly from each region. Initial screening was done on 207 women with a gestational age of 30 to 40 weeks, of whom 96 met the trial criteria and agreed to participate in this study. The inclusion criteria were 18–35 years of age, primigravid, normal singleton pregnancy, and 1+ or 2+ pitting edema in feet. The exclusion criteria were any underlying diseases or history of them (e.g., diabetes, hypertension, preeclampsia, and thrombophlebitis), history of infertility, drug abuse, existence of wound and skin lesions at the massage site, consumption of a specific drug or diet to relieve edema during the study, emergency termination of pregnancy or preterm labor, injury or serious damage in the lower legs, development of preeclampsia during the study, and lack of participation in any massage session and foot circumference assessments. The researcher (MN) explained the aim of the study to the participants. Those who gave written consent were matched and then randomly allocated to one of the three groups (massage with grape seed oil, massage with sweet almond oil, and without intervention). Randomization was done using a random number table generated by a spreadsheet (Excel). The intervention and control groups were matched statistically for age and gestational age, and thus, groups were formed with homogeneous distribution (*P*=0.47 and *P*=0.39, respectively).

The intervention groups received usual health care and a daily 20-minute foot massage with grape seed oil or sweet almond oil. The control group received usual health care. Usual health care included some advices about leg edema such as “avoid hanging your legs during the day” and “when resting, hold your feet above the ground.”

**Figure 1:**
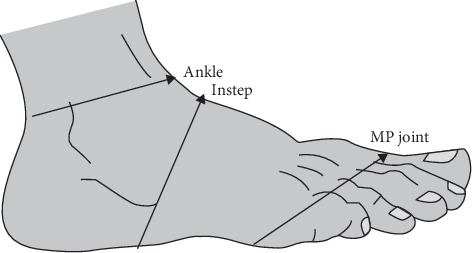
Lower leg circumference measurement (retrieved from the Çoban and Şirin study in 2010). MP joint: metatarsal-phalanges joint.

Participants were interviewed, observed, and examined to take sociodemographic information, medical history, blood pressure, and obstetric characteristics. They were checked for kidney disease with a urine dipstick test. Then, the extent of leg edema was determined by pressing index and middle fingers on the tibia bone for 30 seconds and estimating the depth of the created troughs. After that, circumferences of 3 parts of each leg including the ankle, instep, and foot/toe junction (as shown in Figure 1) were measured by a nonelastic tape measure for each group while the subjects were sitting in an upright position. The ankle circumference was measured medially and laterally above the malleoli, where the diameter was the smallest. The instep circumference was measured over the cuneiform and cuboid bones distal to the heel, and the foot/toe junction circumference was measured at the distal end of the foot, at the metatarsal-phalanges (MP) joint (where the toe joins the foot). The second measurement of the circumferences was done on the fifth day of intervention for the three groups. These measurements were took on an average of 15 minutes. The measurements were done by MR in a private room in the health centres while she was blind about the assignment of people to groups.

The intervention groups were given a 10-minute massage on each foot (a total of 20 minutes of massage for each person) by MN. Before each massage, the researcher washed her hands and made them warm by rubbing them together. Five different movements of massage taken from the Çoban and Şirin study were used in this study [20]. The participants were asked to hold their foot firmly, and then the masseur started massaging following five movements: (1) striking in the entire foot from the toes to the ankle along the top of the foot using the whole hand and returning under the foot to the toes using less pressure, (2) kneading the foot from the toes to the ankle using thumbs while supporting the foot with the fingers underneath, (3) striking the skin surface between each tendon on the top of the foot one after another using thumbs, (4) grasping the foot with both hands and gently manipulating from side to side, and (5) holding the toes with one hand while the other hand supported the foot and the toes were gently bent backward and forward. Each of these movements was performed 10 times in the same order for each foot in turn in each session. The researcher reduced the effects of the masseur's hand fatigue on massaging and pressure on each leg by changing massage sequences in each session. The massage began from the right foot on the first day and from the left foot on the second day and continued in the same way until the fifth day. The massage sessions were held every day at 11 o'clock for 5 days. For convenience of pregnant women, the intervention sessions were held at the participants' houses except the first and the fifth sessions which were held at the health centres. Grape seed oil for one intervention group and sweet almond oil for the other intervention group were used. Each bottle contained 60 cc of sweet almond oil or grape seed oil with a purity of 95% approved by a laboratory. Sweet almond oil and grape seed oil were purchased directly from Barij Essence Pharmaceutical Company and Mahdarou Pharmaceutical Company, respectively.

It should be noted that, before the intervention, massage therapy was taught to the researcher (MN) by a reflexologist, and after the specialist confirmation, the intervention began.

SPSS version 21 was used for statistical analysis. Distribution of the data was tested using the Shapiro–Wilk test. Data are presented as mean ± SD and frequency (percentage). The chi-square, Fisher's exact test, and one-way ANOVA were used to compare proportions between the three groups. Within-group changes were assessed using a paired *t*-test. Between-group differences were assessed using one-way ANOVA and Tukey's test. A *P* value less than 0.05 was considered to be statistically significant.

## 3. Results

**Figure 2:**
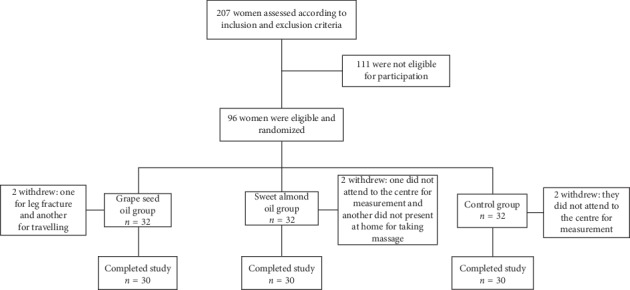
Recruitment and retention of participants in the study.

There were 30 participants in each group. Four participants from the intervention groups and two from the control group withdrew from the study before completing it. The reasons for withdrawal are listed in Figure 2. Descriptive statistics for the three groups' means, standard deviations, and ranges are expressed in Table 1.

The three groups were not significantly different in terms of demographic characteristics, obstetric characteristics, eating habits, activity and rest levels, and amount of fluid and salt intake before the intervention. The results of the average measurements of the participants' left and right ankles, insteps, and MP joints in the intervention and control groups before and after the intervention are shown separately in Table 2. The paired *t*-test showed a significant difference in the intervention and control groups before and after the intervention in terms of the average circumferences of ankles, insteps, and MP joints for both legs. Results of evaluating the measured circumferences of both legs of each participant during two consecutive measurements indicated reduction of the sizes in the two intervention groups and their increase in the control group.

Results of the one-way ANOVA test showed a significant difference between the three groups in terms of the average changes in the measured circumferences (Table 3). Tukey's test that ran to test pairwise comparisons among means indicated significant differences between both intervention groups with the control group. However, the difference was not significant between the two intervention groups.

## 4. Discussion

The results of this study showed a significant difference between the average changes in the measured circumferences of feet in the three groups: the grape seed oil group, the sweet almond oil group, and the control group. Tukey's test that ran to compare the groups two by two indicated significant differences between the grape seed oil group and the control group and also between the sweet almond oil group and the control group. However, the difference was not significant between the two intervention groups. This means that massage with both oils led to reduction in the measured circumferences of feet after five days of intervention compared to that before the intervention. As it is shown in Table 3, despite the fact that the difference in the two intervention groups was not statistically significant, the mean reduction of edema in the sweet almond oil group (except right and left ankles) was slightly greater than that in the grape seed oil group. Therefore, the research hypothesis regarding the greater effect of grape seed oil on reducing the amount of edema in comparison with sweet almond oil was rejected.

Rahimikian et al. in a study on the effects of foot massage on physiological edema of pregnancy confirmed the effectiveness of massage in reducing feet edema. The differences in mean changes in the massage group in the right ankle, left ankle, right instep, left instep, right MPJ, and left MPJ were reported as −0.036, −0.025, −0.020, −0.025, 0.032, and −0.037, respectively [21]. The current study and Rahimikian et al.'s study were similar in terms of the number of days of massage and the method of massage. However, there were some differences between these two studies in the case that Rahimikian et al.'s study compared two groups: massage and control. Plus, they used baby oil for massage. Grape seed oil and sweet almond oil containing antioxidants, vitamins E and B, amino acids, and flavonoids when compared with baby oil, a neutral oil, can be more effective in improving blood flow and consequently reducing edema [23]. Thus, it can be concluded that the more effective the oils used in a massage, the greater the reduction in edema and its resultant discomforts will be.

In another study conducted by Rahimikian et al., regarding the effectiveness of massage and foot elevation in physiological edema, similar results to those of the previous study were obtained [24]. The differences in mean changes in the massage group in the right ankle, left ankle, right instep, left instep, right MPJ, and left MPJ were reported as −0.042, −0.045, −0.042, −0.046, 0.042, and −0.046, respectively. The difference between Rahimikian et al.'s study and the current study was that they had two types of interventions: massage and foot elevation. Also, Rahimikian et al. used baby oil for massaging. The results of this study indicated much more impact of massage on reducing edema, compared to Rahimikian et al.'s study. Massage in the present study was more effective than that in Rahimikian et al.'s study which may be due to differences in geographical location and climatic conditions in the two studies. Rahimikian et al.'s study was conducted in the north of Iran with a warm and humid climate, while the present study was conducted in the southeast of Iran with a warm and dry climate. Humidity causes the fluctuation of body fluids and swelling of tissues [25].

Mollart in a comparative study on the effect of reflexology techniques (lymphatic reflexology and relaxing reflexology) versus rest on the amount of edema showed that rest is more effective than reflexology techniques, focusing on a specific pressure point, in reducing feet edema [22]. The duration of the intervention was 15 minutes per session. The participants' legs were massaged for a few minutes to make them warm before the intervention. Also, grape seed oil was used for massage in the lymphatic reflexology group which was more effective in reducing edema compared to the relaxing reflexology group for whom no oil was used. However, the difference was not significant. The differences in mean changes in the reflexology with grape seed oil group in the right ankle, left ankle, right instep, left instep, right MPJ, and left MPJ were −0.06, −0.10, −0.03, −0.04, 0.03, and −0.29, respectively. These differences were much less compared to the differences in mean changes in the current study. The reasons behind this could be the difference in methods, frequency, and duration of the massage.

Çoban and Şirin in a study on the effect of foot massage on physiological edema showed a 20-minute foot massage with body oil is effective in reducing feet edema [20]. The Çoban and Şirin study was conducted on 80 pregnant women in five 15-minute sessions and significantly reduced *n* average of every measured area in feet except left leg circumference in the massage group compared to the control group. In the Coban and Şirin study, baby oil was used. Differences in mean changes in the Coban and Şirin study were lower than those in the current study. These differences may be because of applying different types of oils and different massage sequences in the two studies. In the current study, massage began from the right foot on the first day and from the left foot on the second day and continued in the same way until the fifth day. Further reasons for the differences in the amount of edema reduction between the current study (Table 3) and other studies may be more amount of foot edema in women in Zahedan. This may be due to ethnicity [26], prolonged standing and lack of adequate rest, lack of proper diet (excess salt consumption and inadequate intake of fruits and vegetables), lack of exercise, and lower economic levels [27]. Another possible reason for more amount of foot edema in women in Zahedan is the high prevalence of the iron deficiency anemia [28]. Leg edema is one of the clinical signs of anemia [29].

On reviewing the literature, no articles examining the effect of sweet almond oil or other effective oils on edema were found, and most studies dealt with lubricant gel or baby body oil. However, the impressive issue in this study was a very good effect of both grape seed and sweet almond oils on reducing edema. Sweet almond oil and grape seed oil are rich in vitamin E, vitamin B, amino acids, and flavonoids which with their strong antioxidant properties result in repairing damaged vessels and improving environmental blood flow [18]. The study of historical documents shows that massage is the oldest form of physical therapy that has been used by humans. Massaging helps return venous blood flow through the pumping action caused by muscle compression. This may lead to edema reduction in lower extremities [21]. So, it is better to use an effective oil to increase the impact of massage on reducing much more amount of edema in a shorter time.

### 4.1. Limitations of the Study

Lack of access to a number of participants at the designated time in 5 consecutive days was a limitation of this study. Therefore, for these participants, massages were done an hour earlier or later than the first day.

## 5. Conclusion

The findings of this study confirmed the effectiveness of foot massage using grape seed and sweet almond oils to reduce pregnancy physiological edema. Therefore, foot massage with appropriate oils can be used as a useful technique by trained midwives in prenatal care centres or at pregnant women houses. As recommendations for future researches, studying the durability of the massage effect after the massage therapy sessions as well as training pregnant women for doing the massages themselves can be mentioned.

## Figures and Tables

**Table 1:** Demographic characteristics by groups.

Characteristics	Grape seed oil group (*n* = 30)		Sweet almond oil group (*n* = 30)		Control group (*n* = 30)	
	Mean	SD	Mean	SD	Mean	SD
Age (years)	25.3	4.06	26.7	5.01	25.63	4.77
Gestational age (weeks)	34.17	2.36	34.01	2.57	33.42	1.59

**Table 2:** Average scores of lower leg circumferences by groups.

Variable			Group					
			Sweet almond		Grape seed		Control	
			Mean ± SD	*n*	Mean ± SD	*n*	Mean ± SD	*n*
The amount of edema before intervention	Right foot	Ankle	23.1 ± 2.5	30	23.1 ± 1.9	30	23 ± 1.6	30
		Instep	23.6 ± 1.7	30	23.6 ± 1.3	30	23.4 ± 1.1	30
		MPJ	23.0 ± 1.3	30	23.0 ± 1.3	30	22.7 ± 1.2	30
	Left foot	Ankle	23.2 ± 2.5	30	23.1 ± 2.0	30	22.9 ± 1.6	30
		Instep	23.6 ± 1.7	30	23.4 ± 1.4	30	23.2 ± 1.2	30
		MPJ	23.0 ± 1.3	30	22.8 ± 1.2	30	22.5 ± 1.0	30

The amount of edema on day 5 after intervention	Right foot	Ankle	22.5 ± 2.4	30	23.5 ± 2.0	30	22.1 ± 1.5	30
		Instep	23.1 ± 1.7	30	23.9 ± 1.5	30	23.0 ± 1.1	30
		MPJ	22.5 ± 1.3	30	23.2 ± 1.4	30	22.2 ± 1.1	30
	Left foot	Ankle	22.5 ± 2.5	30	23.5 ± 2.0	30	22.1 ± 1.6	30
		Instep	23.0 ± 1.6	30	23.7 ± 1.5	30	22.9 ± 1.1	30
		MPJ	22.5 ± 1.3	30	23.1 ± 1.2	30	22.1 ± 1.0	30

MPJ: metatarsal-phalanges joint.

**Table 3:** Differences in mean foot measurements across the three groups (between day 1 and day 5).

Result of ANOVA test	Variable	Group				
		Control		Sweet almond	Grape seed	
Mean measurement changes	Right foot	Ankle	0.396	−0.713	−0.776	*F* = 61.57
						*P*=0.001
		Instep	0.240	−0.460	−0.440	*F* = 28.24
						*P*=0.001
		MPJ	0.266	−0.496	−0.476	*F* = 21.35
						*P*=0.001
	Left foot	Ankle	0.403	−0.770	−0.770	*F* = 50.67
						*P*=0.001
		Instep	0.306	−0.523	−0.366	*F* = 39.59
						*P*=0.001
		MPJ	0.280	−0.440	−0.363	*F* = 17.11
						*P*=0.001

MPJ: metatarsal-phalanges joint.

## Data Availability

No data were used to support this study.

## References

[1] Cunningham F. G., Leveno K. J., Bloom S. L., Spong C. Y., Dashe J. S., Hoffman B. L. (2014). *Williams Obstetrics*.

[2] Ponnapula P., Boberg J. S. (2010). Lower extremity changes experienced during pregnancy. *The Journal of Foot and Ankle Surgery*.

[3] Smyth R. M., Aflaifel N., Bamigboye A. A. (2015). Interventions for varicose veins and leg oedema in pregnancy. *Cochrane Database of Systematic Reviews*.

[4] Saliba J O. A., Rollo H. A., Saliba O., Sobreira M. L. (2019). Graduated compression stockings effects on chronic venous disease signs and symptoms during pregnancy. *Phlebology: The Journal of Venous Disease*.

[5] Spiby H. (1993). Giving complementary therapy with midwifery care for the 1990s. *Midwives Chronicle*.

[6] McCabe P. (1996). Complementary therapy in nursing practice: policy development in Australia. *Australian Journal of Holistic Nursing*.

[7] Tiran D. (1996). The use of complementary therapies in midwifery practice: a focus on reflexology. *Complementary Therapies in Nursing and Midwifery*.

[8] Mitchell M. (2014). Women’s use of complementary and alternative medicine in pregnancy: a search for holistic wellbeing. *Women and Birth*.

[9] Wang M. Y., Tsai P. S., Lee P. H., Chang W. Y., Yang C. M. (2008). The efficacy of reflexology: systematic review. *Journal of Advanced Nursing*.

[10] Watanabe Y., Koshiyama M., Yanagisawa N. (2017). Treatment of leg and foot edema in women. *Women’s Health—Open Journal*.

[11] Richards K. C., Gibson R., Overton-McCoy A. L. (2000). Effects of massage in acute and critical care. *AACN Clinical Issues: Advanced Practice in Acute & Critical Care*.

[12] Enzer S. (2000). *Reflexology: A Tool for Midwives*.

[13] Michalak M. (2018). The use of carrier oils in aromatherapy massage and their effect on skin. *Archives of Physiotherapy & Global Researches*.

[14] Khalifa F. K., Khalil F. A., Barakat H. A., Hassan M. M. (2011). Protective role of wheat germ and grape seed oils in chlorpyrifos-induced oxidative stress, biochemical and histological alterations in liver of rats. *Australian Journal of Basic and Applied Sciences*.

[15] Hulme J., Waterman H., Hillier V. F. (1999). The effect of foot massage on patients’ perception of care following laparoscopic sterilization as day case patients. *Journal of Advanced Nursing*.

[16] Grundy M. M.-L., Lapsley K., Ellis P. R. (2016). A review of the impact of processing on nutrient bioaccessibility and digestion of almonds. *International Journal of Food Science & Technology*.

[17] Chang S. Y. (2008). Effects of aroma hand massage on pain, state anxiety and depression in hospice patients with terminal cancer. *Journal of Korean Academy of Nursing*.

[18] Marzouk T. M. F., El-Nemer A. M. R., Baraka H. N. (2013). The effect of aromatherapy abdominal massage on alleviating menstrual pain in nursing students: a prospective randomized cross-over study. *Evidence-Based Complementary and Alternative Medicine*.

[19] Field T. (2016). Massage therapy research review. *Complementary Therapies in Clinical Practice*.

[20] Çoban A., Şirin A. (2010). Effect of foot massage to decrease physiological lower leg oedema in late pregnancy: a randomized controlled trial in Turkey. *International Journal of Nursing Practice*.

[21] Rahimikian F., Shadmehr A., Mehran A., Kiani M. (2015). Effect of foot massage on physiological edema during pregnancy. *Journal of Knowledge & Health*.

[22] Mollart L. (2003). Single-blind trial addressing the differential effects of two reflexology techniques versus rest, on ankle and foot oedema in late pregnancy. *Complementary Therapies in Nursing and Midwifery*.

[23] Derakhshan S. (2011). Grape seed oil. *Flour and Food Industry Magazine*.

[24] Rahimikian F., Kiani M., Shadmehr A., Kiani M., Kiani M., Niazi Z. (2015). The effectiveness of massage and feet elevation on physiological edema of pregnancy: a comparison. *Payesh*.

[25] Yang B., Li B., Xu C. (2019). Comparison of electrical impedance tomography and intracranial pressure during dehydration treatment of cerebral edema. *NeuroImage: Clinical*.

[26] Xiao J., Shen F., Xue Q. (2014). Is ethnicity a risk factor for developing preeclampsia? An analysis of the prevalence of preeclampsia in China. *Journal of Human Hypertension*.

[27] Montazerifar F., Karajibani M., Sotoudeh M., Amrollahi B. E. (2014). *Evaluation of Nutritional Status in Pregnant Women in Iranshahr*.

[28] Gorgani F., Majlessi F., Momeni M. K., Tol A., Rahimi Foroshani A. (2016). Prevalence of anemia and some related factor in pregnant woman referred to health centers affiliated to Zahedan University of Medical Sciences in 2013. *Razi Journal of Medical Sciences*.

[29] Goonewardene M., Shehata M., Hamad A. (2012). Anaemia in pregnancy. *Best Practice & Research Clinical Obstetrics & Gynaecology*.

